# MeSiC: A Model-Based Method for Estimating 5 mC Levels at Single-CpG Resolution from MeDIP-seq

**DOI:** 10.1038/srep14699

**Published:** 2015-10-01

**Authors:** Yun Xiao, Fulong Yu, Lin Pang, Hongying Zhao, Ling Liu, Guanxiong Zhang, Tingting Liu, Hongyi Zhang, Huihui Fan, Yan Zhang, Bo Pang, Xia Li

**Affiliations:** 1 College of Bioinformatics Science and Technology, Harbin Medical University, Harbin, Heilongjiang 150081, China; 2 Key Laboratory of Cardiovascular Medicine Research, Harbin Medical University, Ministry of Education; 3 Department of Genetics, Harbin Medical University, Harbin 150081, Heilongjiang 150081, China

## Abstract

As the fifth base in mammalian genome, 5-methylcytosine (5 mC) is essential for many biological processes including normal development and disease. Methylated DNA immunoprecipitation sequencing (MeDIP-seq), which uses anti-5 mC antibodies to enrich for methylated fraction of the genome, is widely used to investigate methylome at a resolution of 100–500 bp. Considering the CpG density-dependent bias and limited resolution of MeDIP-seq, we developed a Random Forest Regression (RFR) model method, **MeSiC**, to estimate DNA methylation levels at single-base resolution. MeSiC integrated MeDIP-seq signals of CpG sites and their surrounding neighbors as well as genomic features to construct genomic element-dependent RFR models. In the H1 cell line, a high correlation was observed between MeSiC predictions and actual 5 mC levels. Meanwhile, MeSiC enabled to calibrate CpG density-dependent bias of MeDIP-seq signals. Importantly, we found that MeSiC models constructed in the H1 cell line could be used to accurately predict DNA methylation levels for other cell types. Comparisons with methylCRF and MEDIPS showed that MeSiC achieved comparable and even better performance. These demonstrate that MeSiC can provide accurate estimations of 5 mC levels at single-CpG resolution using MeDIP-seq data alone.

DNA methylation, whereby an enzymatic modification occurs at 5′ position of cytosine pyrimidine ring, plays a central role in cellular processes such as genome regulation and development^1^. In somatic cells, the primary location of 5-methylcytosine (5 mC) is found exclusively in the context of CpG dinucleotide which constitutes only approximately 1% of genome. This modification is generally regarded as a ‘silencing’ epigenetic mark because of the recruitment of methyl-CpG binding proteins^2^. Changes in DNA methylation patterns are closely related with many diseases, particularly cancer^3^. Therefore, profiling DNA methylome is pivotal to understanding the roles of epigenetics in pathogenesis of diseases.

The field of DNA methylation is capitalizing on the sequencing technology. Whole genome bisulfite sequencing (BS-seq)^4^,^5^ is considered as a golden standard to quantify and analyze genome-wide DNA methylation levels at single-base resolution^6^. However, for reason of high cost of BS-seq, it is unfeasible to characterize the methylomes of large biological samples nowadays^7^. Reduced representation bisulfite sequencing (RRBS), which is introduced to reduce sequence redundancy by focusing the sequencing on predefined set of CG-rich regions, covers 2–3 million CpG sites in the human genome^8^. The Infinium HumanMethylation27 microarray is also a widely used platform, which is enable to interrogate more than 27,000 CpG sites from promoters of most protein-coding genes and some microRNAs^9^. Methylated DNA immunoprecipitation sequencing (MeDIP-seq), a popular 5 mC capture-based method, is rapid and cost-efficient to detect genome-wide DNA methylation levels at a resolution of 100–500 bp. This approach requires low-input DNA and has extensive applications in the study of DNA methylation^10^. Recently, the use of MeDIP-seq has produced fruitful outcomes in understanding the role of DNA methylation in cancer genetics and epigenetics. For instance, global hypomethylation was observed as a hallmark of breast cancer^11^ and aberrant DNA methylation was found to play a major role in schizophrenia and bipolar disorder^12^.

Previous studies have proved that MeDIP-seq tends to introduce bias in CpG-poor regions^13^. A number of computational methods and bioinformatics tools have been developed for calibrating the intrinsic bias of MeDIP-seq to accurately estimate 5 mC levels, such as Batman^14^, MEDIPS^15^ and MeDUSA^16^. Batman, a Bayesian deconvolution-based strategy, is used to analyze DNA methylation profiles generated from oligonucleotide arrays (MeDIP-chip) or MeDIP-seq. It proposes the concept of coupling factors which measure the effect of varying densities of methylated CpG sites on MeDIP enrichment and help to accurately quantify methylation levels in windows of fixed size. MEDIPS integrates concepts from BATMAN and MEDME^17^ into MeDIP-seq data analysis pipeline, which weights raw MeDIP-seq signals with coupling factor-based normalization parameters that are identified by linear regression. Different from Batman and MEDIPS, MeDUSA combines numerous software packages to analyze MeDIP-seq data, which mainly focus on the identification of relative changes in DNA methylation (including potential non-CpG methylation) between cohorts. All these methods provide regional normalization of DNA methylation levels. Despite the recent progress, the limited resolution still hampers deeper analysis and interpretation of DNA methylation data. To further increase resolution, a recent approach methylCRF, combines MeDIP-seq and MRE-seq data to assess DNA methylation levels at single-base resolution by employing a Conditional Random Fields algorithm^18^.

In this study, we proposed a model-based methodology, MeSiC (prediction from **Me**DIP-seq data at **si**ngle-**C**pG resolution), that only uses MeDIP-seq data to obtain reliable estimations of 5 mC levels at single-CpG resolution. We found that MeSiC was able to provide accurate estimations of 5 mC levels and calibrate the CpG density-dependent bias. Meanwhile, we further validated the accuracy of MeSiC models using additional biological samples. MeSiC had better performance than MEDIPS at regional levels. When comparison was performed at single-base resolution, MeSiC provided comparable accuracy with methylCRF.

## Results

### Characterization of CpG density-dependent bias in MeDIP-seq data

MeDIP-seq, as a technique for measuring DNA methylation levels with high genome coverage and low cost, has been widely applied in DNA methylome analysis. However, MeDIP-seq biases toward genomic regions with high CpG density and shows deviations in CpG-poor genomic regions^13^,^17^. We therefore examined whether MeDIP-seq signals could be affected by CpG density in human embryonic stem cell line H1.

**Figure 1:**
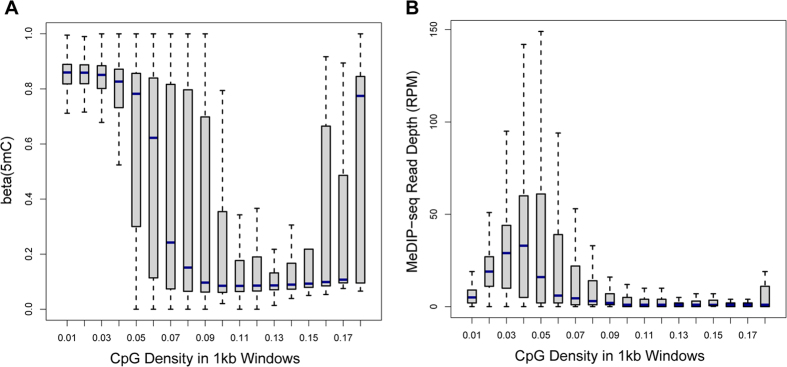
CpG density-dependent bias in MeDIP-seq data. We divided the genome into 1-kb non-overlapping windows and calculated average 5 mC level, RPM value and CpG density for each window. We classified the windows into 18 groups according to CpG density and we compared (**A**) the distribution of 5 mC levels and (**B**) the distribution of RPM values among these groups.

We divided the genome into 1-kb non-overlapping windows. For each window, we calculated the CpG density and a RPM value (reads per million) representing normalized read depth using MeDIP-seq data. Combining BS-seq and TAB-seq data, we calculated single-base resolution 5 mC levels (termed actual DNA methylation level) of 25882669 CpG sites (representing 91.73% of CpG sites in the human genome) by subtracting TAB-seq hits from BS-seq hits. The average 5 mC level of CpG sites within each window was obtained. Through comparing the distribution of 5 mC levels with that of MeDIP-seq derived RPM values, we found that highly methylated CpG sites were mainly enriched for windows with low CpG density but the enrichment was not observed in MeDIP-seq profile (Fig. 1A,B), suggesting the limited accuracy of MeDIP-seq data in low CpG density regions.

### Construction and validation of MeSiC models

MeDIP-seq uses antibody-based immunoprecipitation of 5-methylcytosine to retrieve methylated fragments from sonicated DNA. MeDIP-seq signals represent the methylation level of a certain genomic region rather than an individual CpG site, which are critically dependent on the degree of methylation, local CpG density and relative positions of CpG sites. Moreover, previous studies reported similar methylation levels between neighboring CpG sites^19^,^20^,^21^. Therefore, it is reasonable to assume that MeDIP-seq signals of single CpG sites are contributed by the DNA methylation levels of the CpG site itself and its surrounding CpG sites. That is, the 5 mC level of a certain CpG site can be reflected by the MeDIP-seq read counts of the CpG site itself and its surrounding CpG sites.

**Figure 2:**
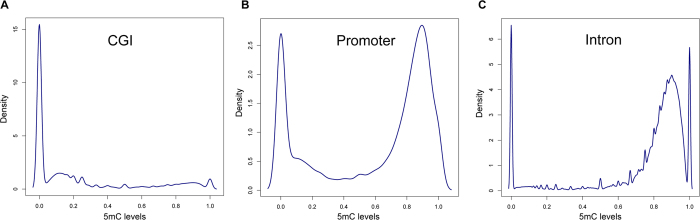
The different distributions of 5 mC levels at different genomic elements. The distribution of 5 mC levels at CGI (**A**), promoter (**B**), intron (**C**).

Based on the above-mentioned assumption, we proposed a method, MeSiC, that used the Random Forest Regression (RFR) algorithm to model 5 mC levels of single CpG sites using MeDIP-seq signals and genomic features. For each CpG site, we selected the 400 bp region centered on the CpG site to calculate a total of 404 features referring to MeDIP-seq signals for single bases, coupling factors (cf), CpG density, GC content and evolutionary conservation. Subsequently, we built MeSiC models using the 5 mC levels and 404 features. Notably, we observed that different genomic elements exhibited different 5 mC distributions. CGI showed a unimodal 5 mC distribution (Fig. 2A), while promoter and intron showed a bimodal (Fig. 2B) and multimodal distribution (Fig. 2C), respectively. Thus, MeSiC trained different models for distinct genomic elements (see Methods).

**Figure 3:**
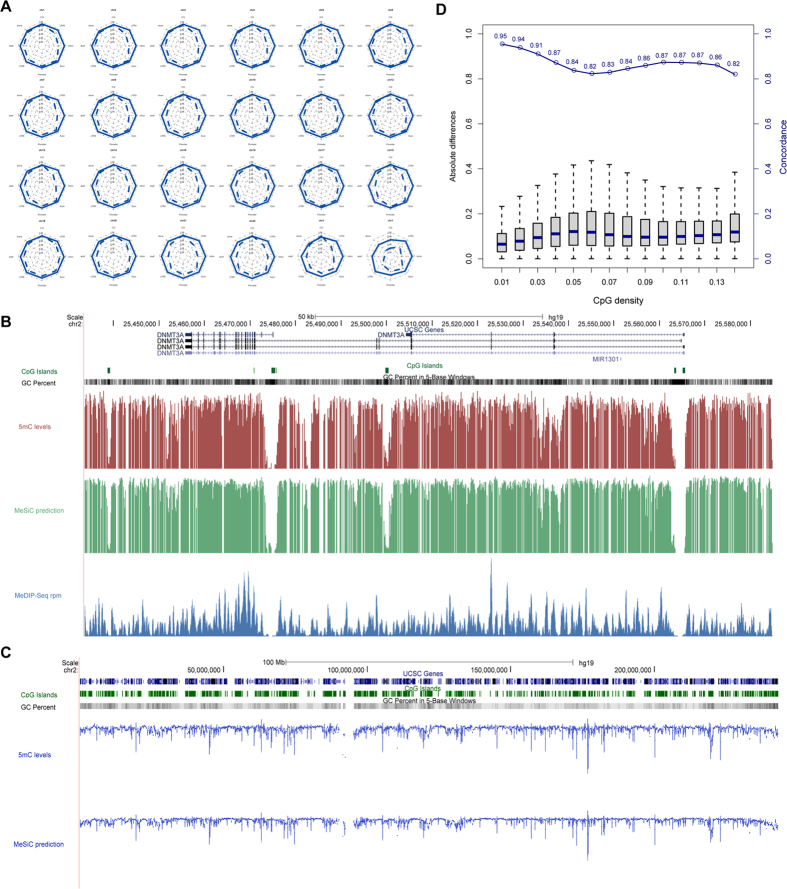
MeSiC enabled to accurately predict 5 mC levels. (**A**) For each genomic element, we evaluated the performance of MeSiC on all 24 chromosomes. (**B**) The UCSC Browser screenshot of *DNMT3A* illustrating 5 mC levels (red), MeSiC predictions (green) and MeDIP-seq reads coverage (blue). (**C**) The UCSC Browser screenshot showing 5 mC levels (top track) and MeSiC predictions (bottom track) in a 100-kb sliding window on chromosome 2. (**D**) The distribution of absolute differences between MeSiC predictions and 5 mC levels in different CpG-density groups.

Chromosome 1, the largest of the human chromosomes, is expected for more representative of genetic properties^22^. Thus, for each genomic element of chromosome 1, its 25% of CpG sites (the ‘training set’) were randomly selected to construct a RFR model that could be used to predict the 5 mC levels of the remaining 75% of CpG sites (the ‘test set’). We used two measurements to evaluate model performance: (i) Pearson correlation coefficient (PCC) between MeSiC predictions and 5 mC levels; (ii) concordance, that is the proportion of CpG sites with absolute methylation difference less than 0.25 between MeSiC predictions and 5 mC levels^23^. For each MeSiC model, we repeated 10 times to generate the training and test sets and the standard deviations of PCCs and concordances were both less than 0.003, indicating that taking 25% of CpG sites of chromosome 1 was enough to construct MeSiC models. We found that MeSiC showed high PCCs and concordances in CGI- and gene-related genomic elements, including CGI, shore, shelf, 5′UTR, promoter, exon, intron and 3′UTR of 24 chromosomes (Fig. 3A). The average PCCs of 24 chromosomes for most genomic elements were around 0.80 with the highest one of 0.85 in CGI. The concordances ranged from 0.83 to 0.92. For example, *DNMT3A* gene encodes an important DNA methyltransferase that catalyzes the transfer of a methyl group to CpG dinucleotide^24^. A genome browser view of actual, predicted 5 mC levels and normalized MeDIP-seq read counts of CpG sites around this gene were shown at single-base resolution (Fig. 3B), and we found a high concordance (0.90) between predictions and 5 mC levels. Moreover, similar results were observed when predictions and 5 mC levels were shown with a 100-kb sliding window on chromosome 2 (PCC and concordance increased to 0.91 and 0.99, respectively; Fig. 3C). In addition, we applied the MeSiC models derived from chromosome 1 to another MeDIP-seq data of the H1 cell line and found a high correlation with pervious predictions (PCC = 0.94, *p-value* < 1.2e^−15^). These results suggested that MeSiC could generate accurate estimation of 5 mC level at single-base resolution. Moreover, we observed that repetitive elements showed low PCCs (Supplementary Figure 1), perhaps due to the unreliable MeDIP-seq signals or mapping errors^25^,^26^. In the following analysis, we mainly focused on the CGI- and gene-related genomic elements which include 12894746 CpG sites (45.7% of all CpG sites in the human genome).

Next, we tested whether MeSiC could correct the effect of CpG density on the measurement of DNA methylation levels for MeDIP-seq data on the whole genome. For each CpG site, we calculated the CpG density of its flanking 500 bp region, based on which we divided CpG sites into different groups. We found that the medians of absolute differences between predictions and 5 mC levels were all less than 0.15, and the concordances were from 0.82 to 0.95 (Fig. 3D). These results demonstrated that the high accuracies of MeSiC models were independent of CpG density.

### Robust performance of MeSiC

**Figure 4:**
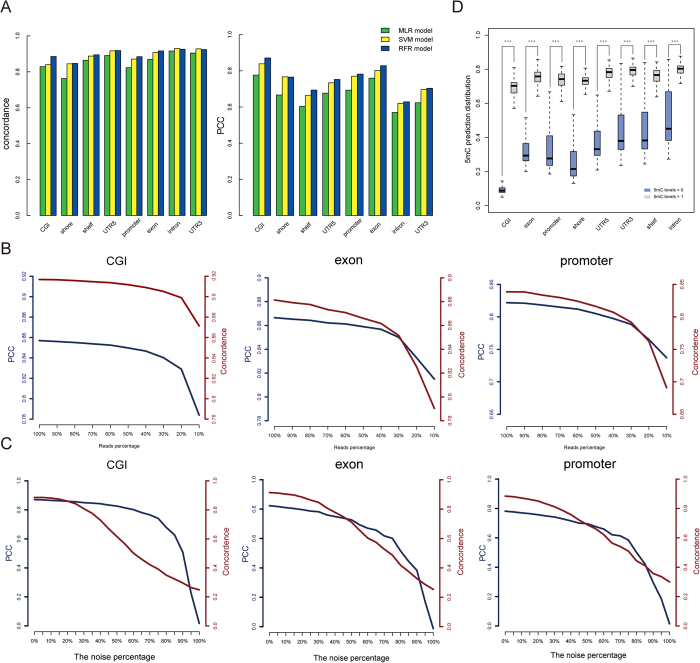
Robust performance of MeSiC. (**A**) We compared concordances (left panel) and PCCs (right panel) between 5 mC levels and predictions of MLR (green), SVM (yellow) and RFR (dark blue) models. The concordances and PCCs between 5 mC levels and predictions of RFR models (**B**) with the percentage of MeDIP-seq reads decreasing from 100% to 10% in decrements of 10% and (**C**) with the percentage of noise increasing from 0 to 100% in increments of 5%. (**D**) The distributions of MeSiC predictions at CpG sites where MeDIP-seq coverages were 0 while the methylation status were either fully methylated (5 mC levels = 1) or fully unmethylated (5 mC levels = 0), and *** indicates *p-value* < 2.2e^−16^.

To evaluate the robustness of MeSiC, we respectively replaced the RFR algorithm with Multivariate Linear Regression (MLR) and Support Vector Regression (SVR) to train different types of models and compared their performances (Fig. 4A). RFR models nearly always showed the highest PCCs and concordances for all genomic elements. Notably, despite some differences, all three types of models obtained good performances, suggesting that the high performance was a direct result of the appropriate features which could comprehensively capture the information of DNA methylation patterns.

It has been demonstrated that sequencing depth is an important factor to impact the stability of MeDIP-seq detection^15^. Subsequently, we examined the effect of MeDIP-seq depth on the model performance. By randomly selecting a decreasing number of MeDIP-seq reads from 100% to 10% in decrements of 10%, we trained multiple models based on various proportions of reads and then calculated the PCCs and concordances between 5 mC levels and MeSiC predictions. We repeated the above process ten times and calculated the average PCC and concordance. We observed relatively stable performance of MeSiC even using 40% of total reads (Fig. 4B), suggesting that the sequencing depth of MeDIP-seq has little effect on MeSiC.

We further evaluated the anti-noise performance of MeSiC through replacing 5 mC levels of a percentage of CpG sites with random values (regarded as noise) when training models (Fig. 4C). With the percentage of noise increasing from 0 to 100% in increments of 5%, there was no distinct difference in prediction performance until the noise percentage was added up to 45%, suggesting that MeSiC models were robust to random noise. Notably, although MeDIP-seq provides extensive coverage of human genome, there are still approximately 30% genomic regions covered by no MeDIP-seq reads^25^. In order to test the performance of MeSiC for distinguishing regions that are truly unmethylated (thus no reads) versus methylated regions that happen to have no reads (stochastic events in genomic experiments, regions that are difficult to assay), we identified two classes of CpG sites (referring to 3650434) with no reads, one showing fully methylation (termed “methylated group”) and the other showing unmethylation (termed “unmethylated group”) on CGI- and gene-related genomic elements. We found that CpG sites in the unmethylated group showed lower MeSiC predictions than those in the methylated group (Wilcox ranked sum test, *p-values* < 2.2e^−16^) in all genomic elements (Fig. 4D). Notably, the CpG sites in the methylated group consistently showed high MeSic predictions despite without read coverage in MeDIP-seq.

### Benchmarking against diverse types of data

#### Validation with different platforms

**Figure 5:**
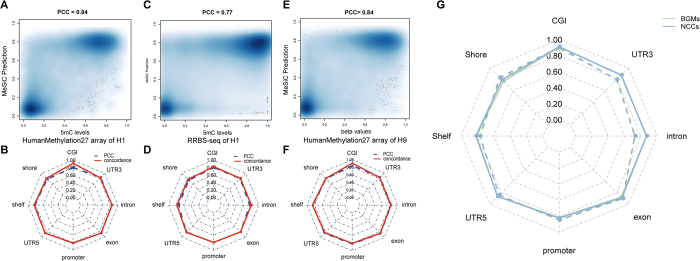
Validation of MeSiC predictions with diverse data. Smoothed color density representation of the scatter plot showing PCCs between MeSiC predictions and (**A**) Infinium HumanMethylation27 microarray data of the H1 cell line, (**C**) RRBS data of the H1 cell line and (**E**) Infinium HumanMethylation27 microarray data of the H9 cell line. For each genomic element, PCCs and concordances relative to (**B**) Infinium HumanMethylation27 microarray data of the H1 cell line, (**D**) RRBS data of H1 cell line and (**F**) Infinium HumanMethylation27 microarray data of the H9 cell line were calculated. Red solid line represents concordances and blue dash line represents PCCs. (**G**) Correlations and concordances of MeSiC relative to BS-seq data for BGMs and NCCs. Green and blue lines represent the results of BGMs and NCCs. Solid and dash lines represent concordances and PCCs, respectively.

Recently, there is a series of DNA methylation techniques which greatly contribute to understanding the biological function of DNA methylation. For example, Infinium HumanMethylation27 microarray is a reliable method to investigate DNA methylation levels at single-CpG resolution and previous studies have confirmed its quantitative accuracy^9^. Reduced representation bisulfite sequencing (RRBS) enables detection and quantification of DNA methylation levels at single-nucleotide resolution^23^. Both Infinium HumanMethylation27 microarray and RRBS can be used to evaluate the accuracy of our models. We directly compared predicted CpG methylation levels of MeSiC models with those from Infinium HumanMethylation27 microarray or RRBS. After data preprocessing and filtering (see Methods), the common detected CpG sites in the H1 cell line were selected for the following analysis. The overall PCC of MeSiC predictions was 0.84 relative to the hydroxymethylation-corrected 27 K data (Fig. 5A). We observed a bimodal methylation distribution of CpG sites, most of which showed high or low methylation and only a small portion of which showed intermediate methylation in both MeSiC predictions and Infinium HumanMethylation27 microarray data. With respect to different genomic elements, we found that their PCCs were in the range of 0.78 to 0.87 and concordances were from 0.81 to 0.92 (Fig. 5B). Moreover, we obtained similar results when comparing MeSiC predictions with hydroxymethylation-corrected RRBS data. The overall PCC was 0.77 (Fig. 5C) and the range was from 0.73 to 0.82 at distinct genomic elements with corresponding concordances from 0.76 to 0.83 (Fig. 5D).

#### Validation with additional samples

To further validate our models, we compared MeSiC predictions for the H1 cell line with Infinium HumanMethylation27 microarray data of the H9 cell line that has similar DNA methylation patterns to the H1 cell line^27^. The overall PCC was 0.84 (Fig. 5E), and PCCs and concordances were from 0.79 to 0.86 and from 0.80 to 0.91, respectively, at distinct genomic elements (Fig. 5F). These findings indicated that the predictions of MeSiC were highly consistent with Infinium HumanMethylation27 microarray data, especially at CGI, shore, 5′UTR and exon, which was analogous to the observations in the H1 cell line.

Next, we sought to investigate whether MeSiC models derived from the H1 cell line could be used to estimate DNA methylation levels for other biological samples. We applied MeSiC models to MeDIP-seq data of Brain Germinal Matrix cells (BGMs) and Neurosphere Cultured Cells Cortex Derived (NCCs). To evaluate our prediction results, we obtained their BS-seq data from the Human Epigenome Atlas and calculated the beta values representing actual DNA methylation levels. The PCCs between predicted values of MeSiC and actual ones were from 0.73 to 0.90 for BGMs and from 0.73 to 0.91 for NCCs at different genomic elements (Fig. 5G). Concordances were from 0.76 to 0.91 for BGMs and from 0.79 to 0.91 for NCCs. These results were similar to those shown in Fig. 3A, nearly reaching accuracy of those from the H1 cell line, suggesting that the H1 cell line-derived models could be applied to other tissue samples or cell types.

### Comparison with other methods

**Figure 6:**
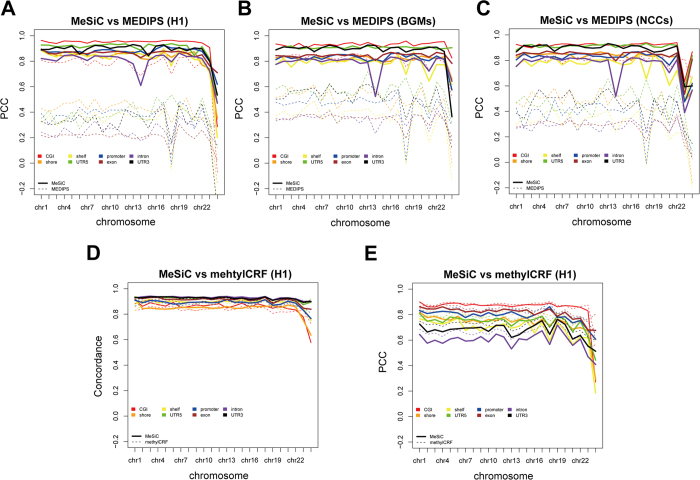
Comparison of the performance of MeSiC with MEDIPS and methylCRF. (**A**) Correlation analysis of MeSiC predictions and MEDIPS-normalized ams relative to 5 mC levels at different genomic elements on 24 chromosomes of the H1 cell line. Correlation analysis of MeSiC predictions and MEDIPS-normalized ams relative to BS-seq values at different genomic elements on 24 chromosomes of (**B**) BGMs and (**C**) NCCs. The ams which corrects for the CpG density of the element was calculated through dividing mean rms by mean coupling factors of bins that located in the element. (**D**) Concordance and (**E**) correlation analysis of the predictions of MeSiC and methylCRF relative to 5 mC levels at different genomic elements on 24 chromosomes of the H1 cell line. Solid and dash lines represent MeSiC and methylCRF, respectively. Different colors represent different genomic elements.

MEDIPS has been widely used to quantify and analyze genome-wide DNA methylation^15^. We compared the performance of MeSiC and MEDIPS (version 1.12.0) at different genomic elements using MeDIP-seq data of the H1 cell line. Since MEDIPS normalizes MeDIP-seq signals in fixed-size window instead of individual CpG site, we calculated the absolute methylation score (ams) for each element. To compare with MEDIPS, we calculated the average MeSiC prediction values and the average 5 mC levels of CpG sites within each element. Because the ams and MeSiC prediction values have different magnitude ranges, we only used PCCs to compare the performance of MeSiC and MEDIPS. On the whole, the PCCs between MeSiC predictions and 5 mC levels were almost all above 0.80, with the highest 0.96 at CGI (Fig. 6A). However, most PCCs between ams and 5 mC levels were from 0.20 to 0.50 except 0.80 for CGI, which were significantly lower than those of MeSiC (one-tailed Student’s t-test, *p-value* = 2.3e^−05^). In addition, we used MeDIP-seq data of BGMs and NCCs to compare the performance of both methods and the similar results with the H1 cell line further validated MeSiC’s superiority (Fig. 6B,C).

Next, we compared MeSiC with methylCRF which predicts DNA methylation levels at single-CpG resolution through integrating MeDIP-seq and methylation-sensitive restriction enzyme sequencing (MRE-seq) data based on Conditional Random Fields algorithm. Because methylCRF provides single-base predictions of DNA methylation levels, we thus directly used the MeSiC single-base predictions to compare with methylCRF. For each genomic element on each of the 24 chromosomes, we calculated the concordances and PCCs of MeSiC and methylCRF relative to 5 mC levels. Both methods showed stable performance. The average concordances were from 0.84 to 0.93 for MeSiC and from 0.83 to 0.92 for methylCRF at different elements (Fig. 6D). The highest concordances were all above 0.91 at intron, 5′ UTR and 3′ UTR for both methods. And the average PCCs ranged from 0.60 to 0.85 for MeSiC and from 0.65 to 0.87 for methylCRF (Fig. 6E). Both MeSiC and methylCRF displayed the highest PCCs at CGI and exon (more than 0.81). Taken together, MeSiC could generate comparable results with methylCRF even using MeDIP-seq data alone.

## Discussion

In this study, we developed a model-based method, MeSiC, which accurately predicts DNA methylation levels at single-base resolution using MeDIP-seq data. Our models calculated 5 mC levels as reference standards through integrating BS-seq and TAB-seq data. MeSiC calibrated CpG density-dependent bias and provided accurate predictions of 5 mC levels at CGI- and gene-related genomic elements. MeSiC turned out to be robust through using distinct algorithms (including MLR, SVR and RFR) and adding noise during model construction. In addition, the accuracy of MeSiC predictions was validated by Infinium HumanMethylation27 microarray and RRBS data. Notably, when applying models derived from the H1 cell line to MeDIP-seq data of BGMs and NCCs, MeSiC predictions also showed a high concordance with actual DNA methylation levels, suggesting that MeSiC could be broadly applied to MeDIP-seq data of other cell types and tissues.

We compared MeSiC with MEDIPS at regional resolution. The comparison result revealed that MeSiC predictions were more in agreement with reference methylation levels. Using MeDIP-seq data alone, MeSiC can generate comparable DNA methylation predictions with methylCRF, which combines both MeDIP-seq and MRE-seq data to estimate DNA methylation levels at single-base resolution. Noteworthy, this makes MeSiC more practical and feasible because of limited samples simultaneously detected by MeDIP-seq and MRE-seq.

**Figure 7:**
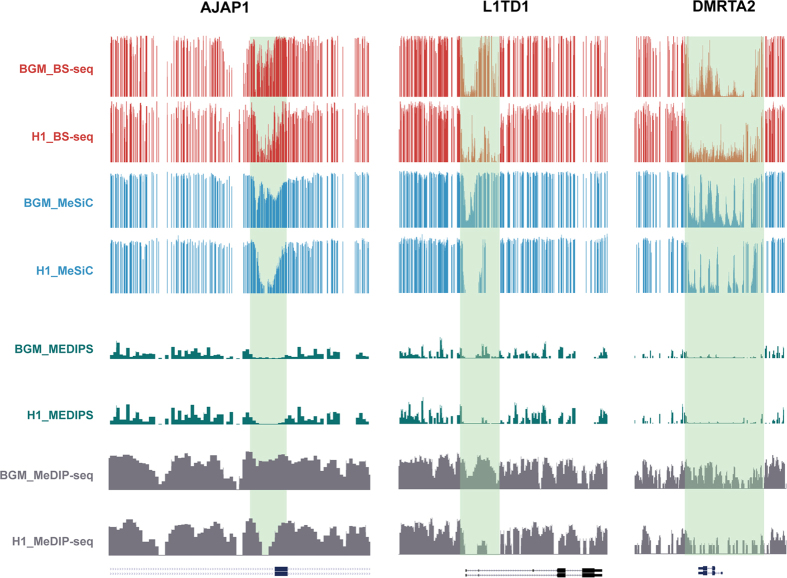
Genome browser views of DNA methylation levels of three gene-related regions in BGMs and the H1 cell line. Red tracks represent beta values of BS-seq. Blue tracks represent predictions of MeSiC. Dark green tracks represent ams of MEDIPS. Gray tracks represent MeDIP-seq read counts (RPKM). Light green shadings represent regions with different methylation levels detected by MeSiC but not MEDIPS.

In addition, we found that the concordance between MeSiC predictions and actual 5 mC levels on the whole genome was 0.93, suggesting that MeSiC could accurately predict 24070882 CpG sites (85.3% of CpG sites in the human genome). Comparison of MeSiC predictions between BGMs and the H1 cell line showed that MeSiC could help us to identify methylation differences (Fig. 7). For example, *AJAP1* has been reported to contribute to the pathogenesis of brain tissues^28^,^29^ and MeSiC predictions showed higher methylation levels in brain tissue than those in the H1 cell line, while we could not observe obvious differences using MEDIPS. *L1TD1*, which is associated with pluripotency of stem cell and cancer cell tumor-propagating capacity^30^,^31^,^32^, also exhibited higher methylation in BGMs and similar results were observed in *DMRTA2*, which plays important roles in the development of the telencephalon in zebrafish^33^ and mammal^34^. Moreover, to explore whether our method can comprehensively capture the differences of DNA methylation levels, we additionally performed a genome-wide identification of differentially methylated regions (DMRs) between BGM and H1 cell line. We first retrieved regions with low PCC (PCC < 0.25) as candidate DMRs using a modified method described by Shaoke Lou^35^. Among these regions, we then identified 1190 DMRs with significant methylation differences using Wilcox ranked sum test. The *p-values* were adjusted by the Benjamini-Hochberg method to control FDR < 0.05. We also found some significantly enriched GO terms of DMR-associated genes, including “positive regulation of excitatory postsynaptic membrane potential”, “N-methyl-D-aspartate receptor (NMDAR) clustering” and “positive regulation of synaptic transmission, glutamatergic”. Since brain functions depend on synergy of both excitatory and inhibitory signal transmission and NMDARs are one of the major subclasses of glutamate receptors which play pivotal roles in synaptic plasticity, brain development and various psychiatric and neurodegenerative diseases^36^, these results suggest that MeSiC also enables to comprehensively detect the brain-related differences of DNA methylation levels between BGM and H1 cell line.

In this study, we considered 5 mC levels rather than BS-seq values as the actual DNA methylation levels to train the models (termed 5 mC-model). In order to explore whether the hydroxymethylation correction is useful for the MeSiC method, we also used BS-seq values to train models (termed BS-model) in the H1 cell line. We calculated the concordances and PCCs of BS-model predictions and 5 mC-model predictions relative to actual 5 mC levels. Overall, the concordances and PCCs of 5 mC-model predictions were higher than those of BS-model predictions for almost all eight genomic elements (Supplementary Figure 2). The average concordance and PCC were 0.862 and 0.737 for BS-model, and 0.895 and 0.753 for 5 mC-model. These results demonstrated that 5 mC-model predictions were more consistent with actual 5 mC levels than BS-model predictions, implicating that hydroxymethylation correction is a necessary step for MeSiC models. Besides, we found that subtracting TAB-seq really made methylCRF predictions better—the PCC increased from 0.72 (uncorrected methylCRF predictions) to 0.80 (corrected methylCRF predictions) and the concordance increased from 0.89 to 0.92, also supporting that it is valid to subtract TAB-seq values from BS-seq values for model construction of MeSiC.

Our results showed the high consistency between MeSiC predictions and 5 mC levels. In addition, we found 25430 common discordant CpG sites (with absolute differences between predictions and references >0.25) on chromosome 1 among the H1 cell line, BGMs and NCCs (Supplementary Figure 3). Considering that SNPs can influence reads alignment^37^,^38^,^39^, we thus examined whether the discordant CpG sites showed a significant enrichment of SNPs (see supplementary information). We found the 25430 common discordant CpG sites were significantly enriched in SNPs (Fisher exact test, *p-value* < 2.2e^−16^; odds ratio, 8.57), suggesting that genetic variants may be one of the potential reasons.

Together, MeSiC permits to accurately estimate DNA methylation levels at single-CpG resolution. It would be a good candidate for quantifying 5 mC levels for MeDIP-seq or other enrichment-based techniques such as MBD-seq. We also provided a web server, which is freely available at http://biocc.hrbmu.edu.cn/MeSiC, to estimate 5 mC levels at single-CpG resolution from MeDIP-seq data for quantitative, high-resolution DNA methylome analysis.

## Materials and Methods

### Data

BS-seq and MeDIP-seq data of the H1 cell line, Brain Germinal Matrix cells (BGMs) and Neurosphere Cultured Cells Cortex Derived (NCCs), and RRBS data of the H1 cell line were obtained from The Human Epigenome Atlas. TAB-seq data of the H1 cell line with pileup format was obtained from Gary C. Hon. Infinium HumanMethylation 27 microarray data of the H1 and H9 cell line were downloaded from GEO datasets. Detailed information of datasets was provided in Supplementary Table 1.

Known genes, CpG islands (CGI), human repetitive elements, phastCons scores and HapMap SNPs were downloaded from the UCSC genome browser^40^. According to the genomic locations of CpG sites, we classified all CpG sites into12 genomic element-related categories according to the annotation of CGI, UCSC known genes and repeat sequences. These 12 categories include CGI, shore (CGI +2 kb/−2 kb), shelf (shore +2 kb/−2 kb), UTR5, promoter (TSS +2 kb/−1 kb), exon, intron, UTR3, SINE, LINE, LTR and others (the remaining CpG sites).

### Data preparation and quality control

According to the description of workflow and analysis pipeline from Epigenomes Data analysis and Coordination Center (EDACC), reads from BS-seq, MeDIP-seq and RRBS data were aligned to the human genome (hg19) using Bowtie software^41^. Only the nonredundant, uniquely mapped reads were used for subsequent analysis.

MeDIP-seq reads were extended to 200 bp (the average fragments size). For RRBS data, we calculated beta values of single CpG sites using BEDTools software^42^. For the HumanMethylation27 microarray data, probes with *p-value* ≥ 0.05 were removed. In addition, we mapped coordinates of CpG sites detected by HumanMethylation27 microarray and TAB-seq from hg18 to hg19 with UCSC genome browser software liftOver utility, respectively.

Generally, BS-seq is considered as the gold standard for the detection of cytosine methylation levels. However, it detects signals that are mixture of 5 mC and 5-hydroxymethylcytosine (5 hmC) and cannot distinguish them. TAB-seq, a Tet-assisted bisulfite sequencing strategy, can accurately detect genome-wide 5 hmC levels at single-base resolution. To obtain actual 5 mC levels for the H1 cell line, we combined BS-seq and TAB-seq as described by Miao *et al.*^43^. Briefly, we calculated BS-seq value and TAB-seq value for each cytosine, respectively. For BS-seq, beta values were calculated by (*N*_*c*_/(*N*_*c*_ + *N*_*t*_)), where *N*_*c*_ represents the number of “C” base as methylated from BS-seq reads and *N*_*t*_ represents the number of “T” base as unmethylated. For TAB-seq, we used (*N*_*hc*_/(*N*_*hc*_ + *N*_*ht*_)) to calculate beta value for each cytosine, where *N*_*hc*_ represents the number of “C” base as hydroxymethylated from TAB-seq reads and *N*_*ht*_ represents the number of “T” base as unhydroxymethylated. Next, in order to ensure the validity of beta values, we limited the analysis to CpG sites with only the ≥4 × BS-seq and TAB-seq read coverage. Then, the binomial distribution B(*N*, *p*) was used to access false positive rates of beta values, where *N* is (*N*_*c*_ + *N*_*t*_) for BS-seq or (*N*_*hc*_ + *N*_*ht*_) for TAB-seq and *p* is the non-conversion rate (0.5% for BS-seq and 2.2% for TAB-seq). Using 1% as the threshold of false discovery rate for both BS-seq and TAB-seq, we identified 25882669 common detected CpG sites. Finally, the 5 mC levels were calculated by subtracting TAB-seq values from BS-seq values for these CpG sites. The genome-wide distribution of 5 mC levels showed significant differences from BS-seq hits (Wilcox ranked sum test, *p-value* < 2.2e^−16^; Supplementary Figure 4).

Besides, to compare with MeSiC, we subtracted 5 hmC levels from methylCRF predictions as the predicted 5 mC levels of methylCRF in the H1 cell line.

### Signal- and genome-related features

For a CpG site *s*, the feature region was defined as the region of 2 mbp around *s* with m-1 bp upstream and m bp downstream (m represents the half of the average fragment size, default = 200). For the *i*^*th*^ base (*i* from 1 to 2 m) in a certain feature region, we calculated its MeDIP-seq signal *x*_*i*_. If the *i*^*th*^ base was cytosine residue within the CpG dinucleotide, *x*_*i*_was equal to RPM value (n), otherwise *x*_*i*_ was 0. As shown in the following equation (1), we could obtain 400 signal features for each CpG site:


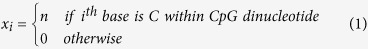


Another feature was coupling factors (cf)^14^ which integrated both MeDIP-seq signal and genomic information. Here, we used cf to weight each base in the feature region by its distance to *s*. It would be calculated as equation (2):


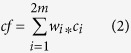


where *w*_*i*_ and *c*_*i*_ are calculated by equations (3) and (4):


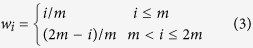



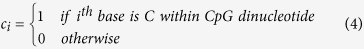


where *w*_*i*_ is the weight of each base within the feature region, which is between 0 and 1. And *c*_*i*_ is 1 when the *i*^*th*^ base is cytosine residue within the CpG dinucleotide, otherwise *c*_*i*_ is 0.

In addition, three genome-related features were taken into consideration, including GC content, CpG density and evolutionary conservation for each feature region. GC content and CpG density were calculated using ‘nucBed’ function of BEDTools and evolutionary conservation was the average phastCons scores of all bases within the feature region.

In total, 404 features for each CpG site were obtained.

### Generation of random forest regression model

Random forest is an ensemble method which combines many classification or regression trees^44^. During the past decade, random forest algorithm was widely used in the field of bioinformatics, such as transcriptional regulation^45^ and drug screen^46^. Random forest can cope with a large set of correlated variables as well as complex interaction structures and it has already shown excellent performance without any tuning parameters^47^. In random forest, two methods are used to ensure the models to reject randomness: (i) Bootstrap aggregation, that is, each tree in the forest is constructed based on a set of randomly selected samples from the training cases (default to 70% for regression). (ii) Random Subspace Method, that is, a small group of input variables are selected at random, at each node to split (default to 30% for regression). The final decision is generated by majority voting from aggregation of the predictions of all trees.

To estimate 5 mC levels at single-CpG resolution from MeDIP-seq data, we used 404 features to build a random forest regression (RFR) model for each genomic element in the H1 cell line. The CpG sites with at least one MeDIP-seq read mapping to the feature region were retained for the following analysis. For the regression model of each genomic element, we randomly selected 25% of CpG sites as training set and the remaining CpG sites were used as test set. A forest of 500 trees was fitted on each genomic element to predict response variable (5 mC levels). For a CpG site shared by multiple genomic elements, we calculated the average of predictions as the final MeSiC prediction.

The random forest regression was implemented using the R package “randomForest” (version: 4.6–7).

## Additional Information

**How to cite this article**: Xiao, Y. *et al.* MeSiC: A Model-Based Method for Estimating 5mC Levels at Single-CpG Resolution from MeDIP-seq. *Sci. Rep.* **5**, 14699; doi: 10.1038/srep14699 (2015).

## Supplementary Material

Supplementary Information
