# Supplementary material for: MeSiC: A Model-Based Method for Estimating 5 mC Levels at Single-CpG Resolution from MeDIP-seq
Source: Sci Rep. 2015 Oct 1;5:14699. doi: 10.1038/srep14699 (PMC4589794; doi:10.1038/srep14699)
Supplement: Supplementary Information [file srep14699-s1.doc]

**MeSiC: A Model-Based Method for Estimating 5mC Levels at** **Single-CpG Resolution from MeDIP-seq**

Yun Xiao^1,2,4^, Fulong Yu^1,4^, Lin Pang^1,4^, Hongying Zhao^1^, Ling Liu^1^, Guanxiong Zhang^1^, Tingting Liu^1^, Hongyi Zhang^1^, Huihui Fan^1^, Yan Zhang^1^, Bo Pang^3^, Xia Li^1,*^

^1^ College of Bioinformatics Science and Technology, Harbin Medical University, Harbin, Heilongjiang 150081, China.

^2^ Key Laboratory of Cardiovascular Medicine Research, Harbin Medical University, Ministry of Education

^3^ Department of Genetics, Harbin Medical University, Harbin 150081, Heilongjiang 150081, China.

^4^ These authors contributed equally to this work.

^*^ Corresponding Author

Email: [lixia@hrbmu.edu.cn](mailto:lixia@hrbmu.edu.cn)

**Supplementary Information**

Gregory R. Grant et al reported that the presence of sequencing errors or SNPs may lead to the alterations of the read sequences and further affect the read alignment[^1^](#_ENREF_1). Yuan Ji et al demonstrated that hidden nucleotide variations, such as a mutation or SNP, is an important source of mapping error that short reads are typically mapped to the public reference genome instead of the sample genome[^2^](#_ENREF_2). Peter Tonner et al reported that SNPs could potentially lead to mismatches of RNA-seq[^3^](#_ENREF_3). Based on these studies, we explored whether the common discordant CpG sites were affected by SNPs. In our analysis, the number of common predicted CpG sites on chromosome 1 among H1 cell line, BGMs and NCCs was 1300626, 0.4% of which contained polymorphisms. We found the 25430 common discordant CpG sites were significantly enriched in SNPs (up to 2.9%) compared with the concordant sites (Fisher exact test, *p-value* <2.2e-16; odds ratio, 8.57).

**Supplementary Figures and Table**

**Figure S1**

**
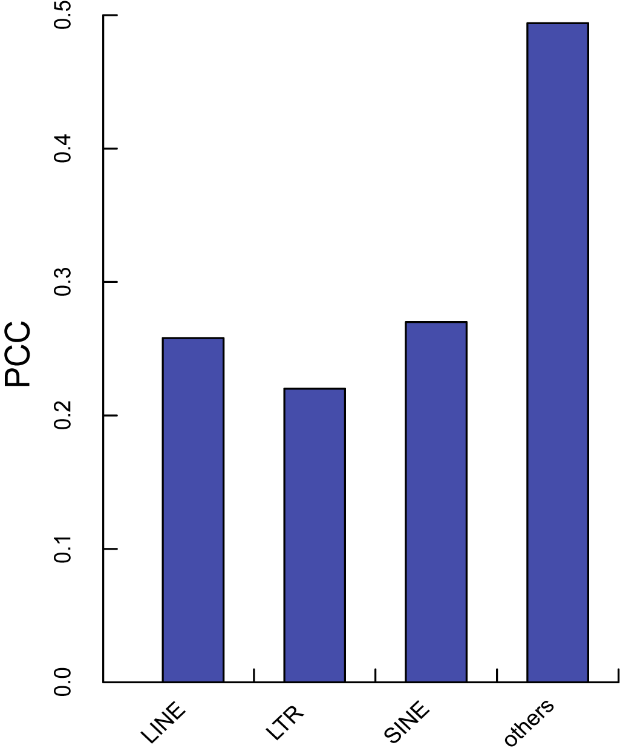
**

**Figure S2**

**
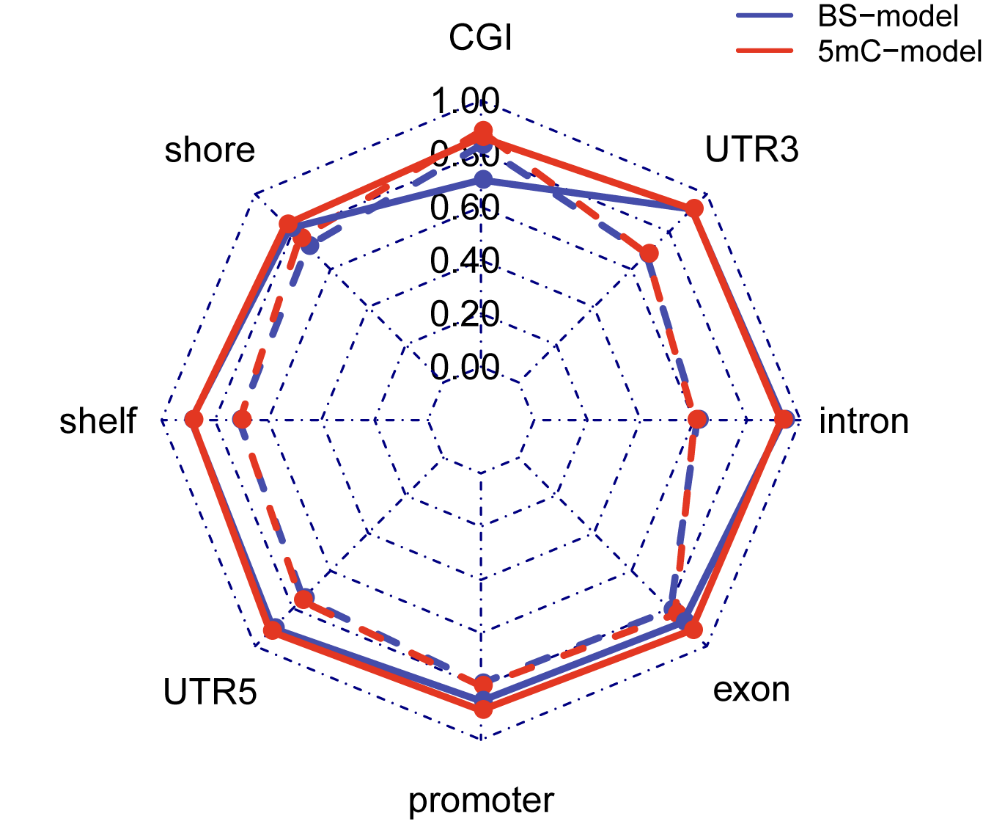
**

**Figure S3**

**
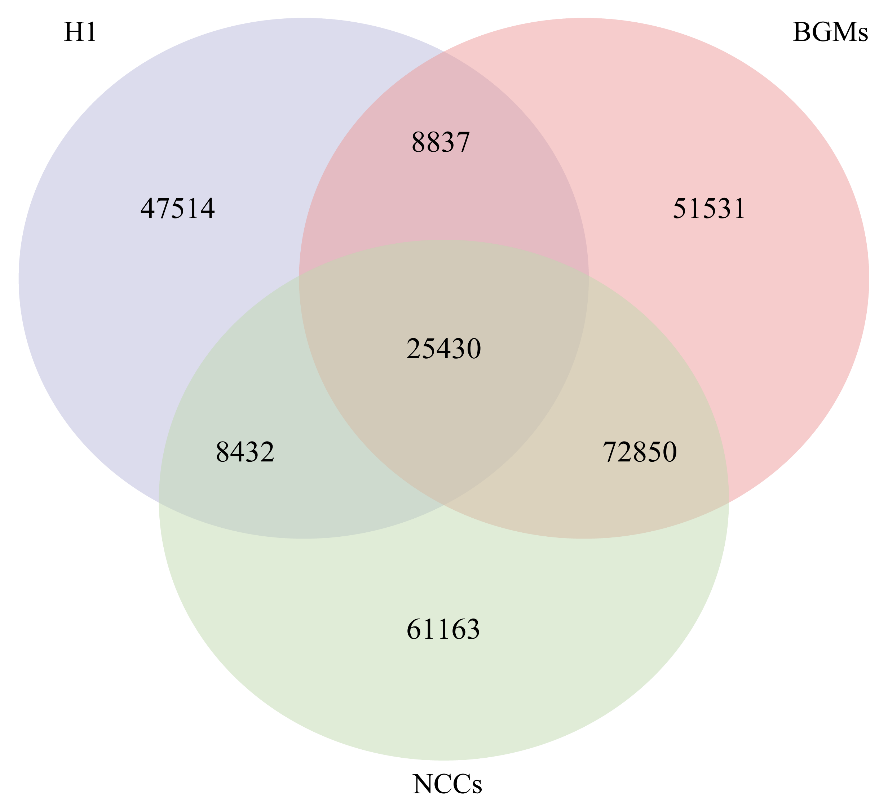
**

**Figure S4**

**
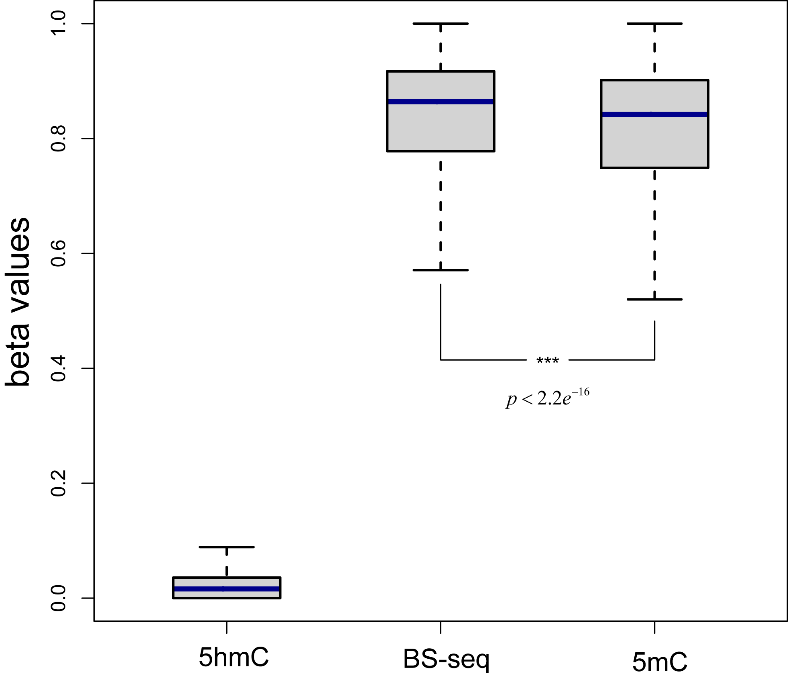
**

**Supplementary Table 1**

| **Sample** | **Technology** | **Accession** |
| --- | --- | --- |
| H1 cell line | BS-seq^1^ | GSM429321 |
|  | TAB-seq^1^ | From Gary C. Hon |
|  | MeDIP-seq^1^ | GSM543016 |
|  | MeDIP-seq (replicate） | GSM456941 |
|  | Human Methylation 27 | GSM739949 |
|  | RRBS | GSM621705 |
| H9 cell line | Human Methylation 27 | GSM1031160 |
| Brain Germinal Matrix  (BGMs) | MethylC-seq | GSM941747 |
|  | MeDIP-seq | GSM707023 |
| Neurosphere Cultured Cells  (NCCs) | MethylC-seq | GSM17129 |
|  | MeDIP-seq | GSM5515 |

^1^ training model

Supplementary Table 2

|  | SNP | NOT SNP |
| --- | --- | --- |
| Discordance | 738 | 24692 |
| Concordance | 4431 | 1270765 |

Fisher’s exact test for given probabilities

*p-value* < 2.2e-16; odds ratio=8.57

**Supplementary Figure/Table Legends**

Supplementary Figure 1. The PCCs between MeSiC predictions and 5mC levels at 4 genomic elements including LINE, LTR, SINE and Others.

Supplementary Figure 2. Comparison of PCCs and concordances between 5mC-models and BS-models at CGI- and gene-related genomic elements. Solid lines represent concordance and dashed lines represent PCC.

Supplementary Figure 3. Recurrence of discordant CpG sites among three biological samples. Venn diagram showing the overlap of discordant CpG sites among the H1 cell line, BGMs and NCCs.

Supplementary Figure 4. The distributions of beta values of 5hmC, BS-seq data and 5mC.

Table S1. Detailed information of datasets used in this study.

Table S2. The common discordant CpG sites were significantly correlated with the occurrence of SNPs.

1 Day, D. S., Luquette, L. J., Park, P. J. & Kharchenko, P. V. Estimating enrichment of repetitive elements from high-throughput sequence data. *Genome biology* **11**, R69, doi:10.1186/gb-2010-11-6-r69 (2010).

2 Ji, Y. *et al.* BM-map: Bayesian mapping of multireads for next-generation sequencing data. *Biometrics* **67**, 1215-1224, doi:10.1111/j.1541-0420.2011.01605.x (2011).

3 Tonner, P., Srinivasasainagendra, V., Zhang, S. & Zhi, D. Detecting transcription of ribosomal protein pseudogenes in diverse human tissues from RNA-seq data. *BMC genomics* **13**, 412, doi:10.1186/1471-2164-13-412 (2012).
